# Subthalamic nucleus stimulation does not influence basal glucose metabolism or insulin sensitivity in patients with Parkinson's disease

**DOI:** 10.3389/fnins.2014.00095

**Published:** 2014-05-06

**Authors:** Nicolette M. Lammers, Brigitte M. Sondermeijer, Th. B. (Marcel) Twickler, Rob M. de Bie, Mariëtte T. Ackermans, Eric Fliers, P. Richard Schuurman, Susanne E. La Fleur, Mireille J. Serlie

**Affiliations:** ^1^Department of Endocrinology and Metabolism, Academic Medical CenterAmsterdam, Netherlands; ^2^Department of Vascular Medicine, Academic Medical CenterAmsterdam, Netherlands; ^3^Department of Diabetology, Endocrinology and Metabolic Diseases, Antwerp University HospitalAntwerp, Belgium; ^4^Department of Neurology, Academic Medical CenterAmsterdam, Netherlands; ^5^Laboratory of Endocrinology, Department of Clinical Chemistry, Academic Medical CenterAmsterdam, Netherlands; ^6^Department of Neurosurgery, Academic Medical CenterAmsterdam, Netherlands

**Keywords:** deep brain stimulation, subthalamic nucleus, insulin sensitivity, dopamine, glucose metabolism

## Abstract

Animal studies have shown that central dopamine signaling influences glucose metabolism. As a first step to show this association in an experimental setting in humans, we studied whether deep brain stimulation (DBS) of the subthalamic nucleus (STN), which modulates the basal ganglia circuitry, alters basal endogenous glucose production (EGP) or insulin sensitivity in patients with Parkinson's disease (PD). We studied 8 patients with PD treated with DBS STN, in the basal state and during a hyperinsulinemic euglycemic clamp using a stable glucose isotope, in the stimulated and non-stimulated condition. We measured EGP, hepatic insulin sensitivity, peripheral insulin sensitivity (Rd), resting energy expenditure (REE), glucoregulatory hormones, and Parkinson symptoms, using the Unified Parkinson's Disease Rating Scale (UPDRS). Basal plasma glucose and EGP did not differ between the stimulated and non-stimulated condition. Hepatic insulin sensitivity was similar in both conditions and there were no significant differences in Rd and plasma glucoregulatory hormones between DBS *on* and DBS *off.* UPDRS was significantly higher in the non-stimulated condition. DBS of the STN in patients with PD does not influence basal EGP or insulin sensitivity. These results suggest that acute modulation of the motor basal ganglia circuitry does not affect glucose metabolism in humans.

## Introduction

There is ample evidence that the central nervous system (CNS) regulates glucose homeostasis. In different neural pathways within the CNS, several neuropeptides and neurotransmitters have been identified that influence insulin sensitivity (Williams et al., 2001; Schwartz and Porte, 2005; Sandoval et al., 2009).

Neuromodulation of specific areas of the brain with deep brain stimulation (DBS) or transcranial direct current stimulation (tDCS) offer unique opportunities to examine the direct role of the CNS in glucose metabolism. Indeed, it has been shown that tDCS promotes systemic insulin sensitivity (Binkofski et al., 2011). DBS of the subthalamic nucleus (STN) is used for the treatment of Parkinson's disease (PD) (Krack et al., 2003).

Besides the beneficial effects on Parkinson symptoms, weight gain is a common side effect of STN DBS and this might be related to reduced energy expenditure due to decreased locomotor activity (Rieu et al., 2011), decreased resting energy expenditure (REE) (Perlemoine et al., 2005) or an increase in energy intake through an effect on brain areas involved in the regulation of body weight (Perlemoine et al., 2005). It has been proposed that the STN DBS-induced current reaches hypothalamic nuclei and hence the effects on energy metabolism might be indirect since the hypothalamus controls food intake and energy expenditure (Sauleau et al., 2009). Furthermore, it has been shown that weight gain is associated with the contact site of the electrodes implanted with STN stimulation, where weight gain is most prominent when the contact sites are in closest relation with the wall of the third ventricle (Rùžièka et al., 2012). The third ventricle is bounded by the hypothalamus on both sides.

In addition to food intake and energy expenditure, the hypothalamus is involved in the regulation of glucose metabolism and insulin sensitivity (Obici et al., 2002; Kalsbeek et al., 2004; Yi et al., 2010) and STN DBS might therefore also affect glucose fluxes. Studies on glucose metabolism in patients with PD and DBS are scarce. One study showed a reduction in endogenous glucose production (EGP) with STN DBS (Batisse-Lignier et al., 2013).

STN DBS modulates the basal ganglia circuitry, where it exerts differential effects on different nuclei (Meissner et al., 2005; Reese et al., 2011). It has been postulated that STN DBS also activates surviving nigrostriatal neurons projecting to the striatum and thereby changing local dopamine concentrations (Marani et al., 2008). Several studies in animals have shown an increase in striatal dopamine with STN DBS (Meissner et al., 2003; Shon et al., 2010). Dopamine is known to be involved in glucose metabolism since dopamine antagonists induce insulin resistance in clinical studies, while dopamine agonists improve glucose intolerance (Pijl et al., 2000; Liebzeit et al., 2001). This seems at least in part to be a centrally regulated effect while drug naïve schizophrenic patients, who have a disturbed central dopaminergic homeostasis, display hepatic insulin resistance compared to healthy controls (Van Nimwegen et al., 2008). Furthermore, animal studies revealed that icv bromocriptine has an effect on glucose metabolism (Luo et al., 1999). In humans, the direct role of central dopamine in the regulation of glucose metabolism and insulin sensitivity is unknown.

We aimed to investigate the effects of STN DBS on glucose metabolism and insulin sensitivity and measured basal glucose metabolism and insulin sensitivity, using a hyperinsulinemic euglycemic clamp with a stable glucose isotope tracer, in patients with PD and DBS of the STN in the stimulated and non-stimulated condition.

## Materials and methods

### Patients

We included men with stable PD, treated with bilateral DBS of the STN. Patients were recruited from the Outpatient Clinic of the Department of Neurology of the Academic Medical Center, Amsterdam, the Netherlands. Exclusion criteria were: age below 18 years, other functional stereotactic neurosurgical interventions (e.g., pallidotomy), unstable weight, psychosis, depression, alcoholism, dyslipidemia (primary or secondary form), use of lipid lowering drugs, use of medication influencing glucose metabolism, (except dopamine agonists), type II diabetes mellitus (DM), first degree family member with type II DM, active smoking, renal insufficiency (creatinine >150 μmol/L) or elevated liver enzymes.

This study was approved by the institutional review board of the Academic Medical Center. Written informed consent was received from all patients prior to inclusion and after the purpose of the study was described.

### Hyperinsulinemic euglycemic clamp

[6,6-^2^H_2_]glucose (>99% enriched; Cambridge Isotopes, Andover, USA) was used to measure EGP based on the principle of the isotope dilution technique.

After an overnight fast the patients were admitted at the Metabolic Unit at 0830 h. For the infusion of stable isotope tracer, insulin, and glucose, a catheter was placed into an antecubital vein of the left hand. For drawing arterialized venous blood another catheter was inserted into a vein of the right hand and kept into a thermo-regulated (60°C) plexiglas box. Saline (NaCl 0.9%) was infused at a rate of 50 ml/h to sustain catheter patency. Before the start of the clamp, at *T* = 0 (0900 h), blood samples for background enrichment were taken, whereafter a primed continuous infusion of a stable glucose isotope was started ([6,6-^2^H_2_]glucose at a rate of 0.11 μmol/kg·min, with a priming dose of 8.8 μmol/kg) and continued during the study day. After a 2 h-equilibration period, blood samples were drawn for isotope enrichments and glucoregulatory hormones. Thereafter, the DBS remained either in the on situation or was turned off. This was done in a single blinded way, in random assignment, by the treating neurologist not otherwise involved in the tests. The researcher performing the clamp was not aware of the on or off situation. For the next 3.5 h, blood samples were taken every 30 min for isotope enrichments and glucoregulatory hormones.

Next, a 1-step hyperinsulinemic euglycemic clamp was started, with a continuous infusion of insulin (Actrapid 100 U/ml; Novo Nordisk Farma, Alphen a/d Rijn, the Netherlands) for 2 h and 10 min (20 mU/m^2^ body surface area min). Every 5 min, plasma glucose concentrations were determined and to maintain a plasma glucose level of 5.0 mmol/L, a solution of glucose 20% was infused at a variable rate. Within this solution 1% was enriched with [6,6-^2^H_2_]Glucose to approximate the enrichment values in plasma and thereby minimize alterations in isotopic enrichment due to the infusion of exogenous glucose (Finegood et al., 1987). During the last 25 min of the hyperinsulinemic period, five blood samples were drawn at 5-min intervals for determination of isotope enrichments and glucoregulatory hormones. During the study, the patients were allowed to drink water only.

Within 1 month the same study day was performed with the DBS electrodes either switched on or off (random assignment).

### Body composition and indirect calorimetry

Body composition was measured at the beginning of both study days using bioelectrical impedance analysis (Maltron BF906; Maltron, Rayleigh, UK).

Oxygen consumption (VO_2_)and CO_2_ production (VCO_2_) were measured continuously during 20 min of every hour of the first 3 h and during the final 20 min of the hyperinsulinemic euglycemic clamp by indirect calorimetry using a ventilated hood system (Sensormedics model 2900; Sensormedics, Anaheim,CA). The mean values of VO_2_ and VCO_2_ were used for the calculation of glucose and fat oxidation.

### Unified parkinson's disease rating scale

Every hour, Parkinson symptoms were assessed with the Unified Parkinson's disease Rating Scale (UPDRS) motor section (Gelb et al., 1999) by a specialized PD nurse that was blinded for the DBS settings (on vs. off). The UPDRS is widely used for the clinical evaluation of PD. A higher score denotes more severe PD symptoms (0 = no symptoms, 108 = worst score).

### Analytical procedures

With the use of a Biosen C-line plus glucose analyzer (EKF Diagnostics, Barbleben/Magdeburg, Germany), plasma glucose levels were determined (the glucose oxidase method). [6,6-^2^H_2_]Glucose enrichment was measured as described earlier (intraassay variation: 0.5–1%, interassay variation: 1%, detection limit: 0.04%) (Ackermans et al., 2001). Plasma levels of insulin and cortisol were measured using the Immulite 2000 system (Diagnostic Products Corp., Los Angeles, CA) (insulin; intraassay variation: 3–6%, interassay variation: 4–6%, detection limit: 15 pmol/l and cortisol; intraassay variation: 7–8%, interassay variation: 7–8%, detection limit: 50 nmol/l).Plasma glucagon concentrations were measured with the Linco ^125^I RIA (Linco Research,St. Charles, MO) (intraassay variation: 9–10%,interassay variation: 5–7%, detection limit: 15 ng/l).

### Calculations and statistics

EGP was calculated using the modified forms of the Steele Equations as described previously (Steele, 1959; Finegood et al., 1987). O2 consumption and CO2 production were used to calculate REE, glucose- and fat oxidation rates, as reported previously (Frayn, 1983).

SPSS version 18.0 (SPSS,Chicago, IL, USA) was used for statistical analysis. If measured values were below limit of detection, for the calculations half of the limit of detection value was used.

To investigate the influence of DBS on basal EGP, glucose, glucoregulatory hormones, and REE, linear mixed-effect models were used to analyze group differences during the first 3.5 h after start of the intervention. Baseline values as confounding factor were included in the model as covariates. The dependency of the measurements within the same subject was accounted for by including subject-specific variables. Different covariance structures were explored, and the model with the best fit was used. Interaction terms between groups and group and time-points were used to examine group related differences between the stimulated and non-stimulated condition. For each of the models, the residuals were normally distributed (Wilk-Shapiro's W > 0.90) and showed constant variance. Comparison of the clamp data within subjects,between the stimulated and non-stimulated condition, were done using the Wilcoxon signs rank test.A *p*-value of < 0.05 was considered statistically significant and a *p*-value of < 0.1 was considered as a trend. Data are presented as median (minimum–maximum) or median ± s.e.m.

## Results

### Patient characteristics

We included 8 men who were diagnosed with PD and treated with bilateral STN DBS. Their baseline characteristics are summarized in Table 1. One patient only completed the first study day due to medical problems not related to this study.

**Table 1:** **Patient characteristics**.

**PATIENT CHARACTERISTICS**		
Age (years)	59	[44–65]
Height (cm)	183.5	[173–189]
Weight (kg)	88.5	[67.9–118.6]
BMI (kg/m^2^)	26.45	[22.7–33.2]
Fat mass (%)	31.65	[21.0–39.0]
Lean mass (%)	68.35	[53.7–71.8]

Data expressed as median [min-max].

As for the use of dopaminergic medication: all patients were on levodopa, with an average dose of 4 × 125 mg/day. Four patients also used D2 dopamine agonists, 7 mg/day on average. The patients took their dopaminergic medication at exactly the same time on both study days.

### Basal glucose metabolism

**Figure 1:**
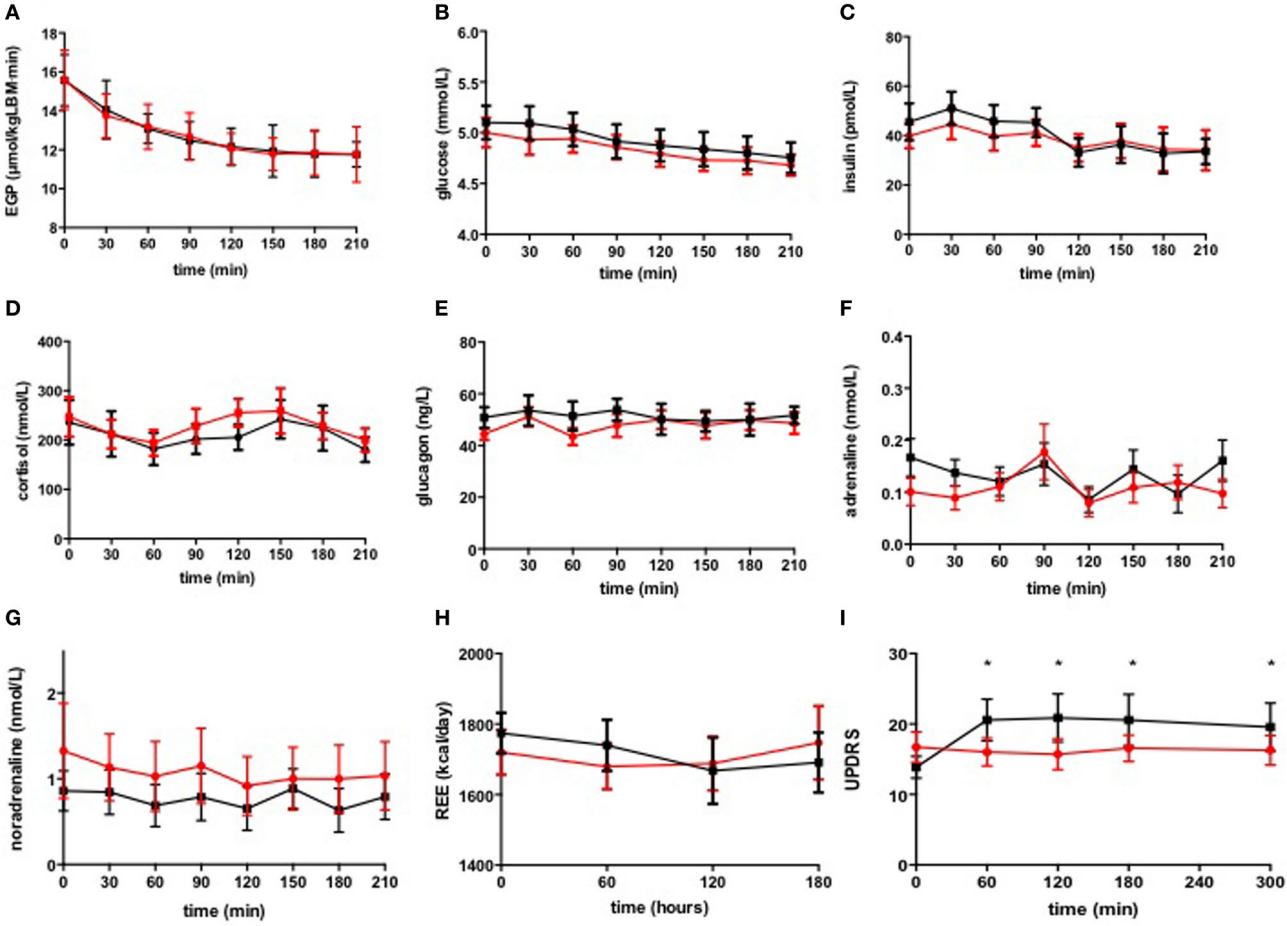
**Basal EGP (A), plasma glucose (B), glucoregulatory hormones (C–G), REE (H), and UPDRS (I)**. DBS switched *on* (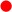) and switched *off* (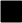). Data are expressed as median ± s.e.m. ^*^p < 0.05.

During the first 3.5 h, basal EGP did not differ between the stimulated and non-stimulated condition (Figure 1A). Plasma glucose and plasma insulin during the first 3.5 h levels were comparable between the two study days (Figures 1B,C).

### Insulin sensitivity

**Figure 2:**
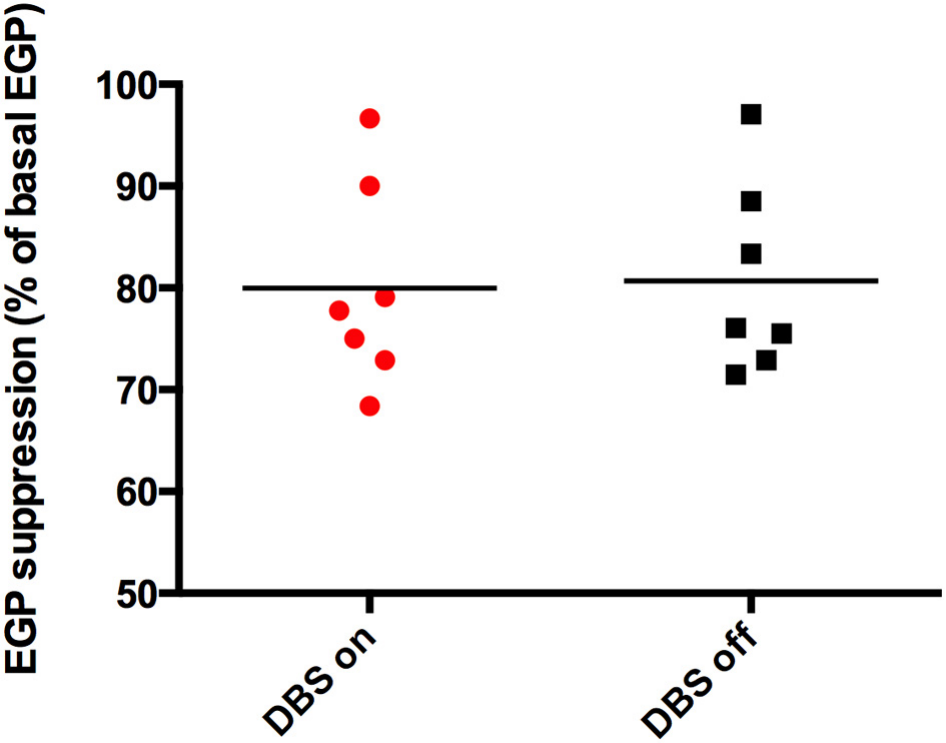
**Hepatic insulin sensitivity**. DBS switched on (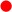) and switched off (◾), *p* = 0.866.

There were no significant differences in plasma concentrations of glucose or insulin during the hyperinsulinemic euglycemic clamps on both study days (Table 2). Insulin-mediated suppression of EGP, expressed as the percentage of basal EGP, was similar in both conditions (stimulated 77.8 [68–97] % vs. non-stimulated 76 [71–97] %, *p* = 0.866) (Figure 2). BMI negatively correlated with hepatic insulin sensitivity (data not shown). Glucose rate of disappearance (Rd) was not significantly different between the two study days (stimulated 14.82 [9.51–21.96] vs. non-stimulated 14.24 [9.06–14.64] μ mol/kg.min, *p* = 0.128).

**Table 2:** **Clamp measurements**.

	**On**	**Off**	***p***
**HYPERINSULINEMIC EUGLYCEMIC CLAMP**			
Glucose (mmol/L)	5.09 [4.8–5.21]	5.01 [4.92–5.13]	0.753
EGP (μ mol/kgLBM·min)	3.40 [0.51–5.31]	3.37 [0.44–5.19]	0.866
Insulin (pmol/L)	191 [149–283]	191 [167–244]	0.176
Glucagon (ng/L)	40 [20–54]	37 [29–62]	0.127
Cortisol (nmol/L)	213 [121–306]	281 [141–342]	0.398
REE (kcal/day)	1718 [1292–1887]	1850 [1352–2030]	0.043

Data are expressed as median [min-max].

### Glucoregulatory hormones

There were no differences in glucoregulatory hormones neither in the basal state nor during the hyperinsulinemic euglycemic clamp, between the stimulated and the non-stimulated condition (Figures 1D–G, Table 2).

### Indirect calorimetry and UPDRS

There were significant differences in REE during the first 3 h between the stimulated and non-stimulated condition. DBS^*^time interaction significantly differed between the 2 groups, *p* = 0.018 (Figure 1H) but DBS^*^group interaction was not significant, suggesting that DBS did not affect basal REE.

During the hyperinsulinemic euglycemic clamp, REE was significantly higher in the non-stimulated condition (REE with DBS on 1718 [1292–1887] vs. with DBS off 1850 [1352–2030] kcal/24 h, *p* = 0.043) (Table 2), although not significantly different when expressed as percentage increase from basal REE.

No differences were found in respiratory quotient (RQ), glucose-, and fat oxidation rates, in the basal state nor in the hyperinsulinemic state (data not shown).

As expected, Parkinson symptoms measured by the UPDRS, were significantly higher when the DBS was switched off during the entire study day (*p* = 0.022, Figure 1I).

## Discussion

We show that STN DBS, in patients with stable PD, does not have an acute effect on basal EGP, hepatic or peripheral insulin sensitivity, nor on basal plasma concentrations of glucose and insulin. Furthermore, STN DBS does not have an effect on other glucoregulatory hormones.

Our results seem in contrast with an earlier report on lower EGP upon STN DBS, i.e., Batisse-Lignier et al showed that basal EGP was decreased in the stimulated condition (Batisse-Lignier et al., 2013). However, the 22% decrease in EGP in that study could alternatively be explained by the physiological adaptation to fasting since EGP was measured in both the non-stimulated and stimulated condition, consecutively on one study day, and thus with a difference in hours of fasting. In fact, the median decrease in EGP in our patients over time was 24% and comparable between the on and the off situation (Figure 1A), representing a physiological decrease in EGP during prolonged fasting.

In our study, there were no significant changes in REE, which could be attributed to the DBS itself. Also, we found no correlation between PD symptoms and REE (data not shown). Regulation of basal energy expenditure strongly depends on hypothalamic control and hypothalamic integration of metabolic signals. Whether these neuronal pathways are different in the stimulated vs. non-stimulated condition is unknown. Long-term studies on energy expenditure in STN DBS treated patients however, all show a decrease in basal and total energy expenditure (decrease of approximately 10%), which in some studies is also explained by a reduction in muscle tone (Rieu et al., 2011). During hyperinsulinemia, REE was significantly lower in the stimulated condition, but when expressed as percentage increase from basal REE these differences were no longer apparent. This suggests that the increase in REE induced by metabolic handling of the infused glucose is not affected by DBS of the STN.

Anatomically, the STN receives input from the globus pallidus and sends excitatory projections to the globus pallidus and the basal ganglia. PD is characterized by a progressive loss of nigrostriatal dopaminergic neurons and consequently leads to a reduced globus pallidus output and an activated discharge from the STN to the basal ganglia. STN DBS modulates the basal ganglia circuitry, where it exerts differential effects on different nuclei. For instance, it has been shown that STN DBS decreases STN activity and enhances pallidal firing rate (Meissner et al., 2005; Reese et al., 2011), with a subsequent altered basal ganglia output. However, not all mechanisms by which STN DBS decreases Parkinson symptoms have been clarified yet. It has been proposed by some researchers that STN DBS also activates surviving nigrostriatal neurons projecting to the striatum, probably via STN—substantia nigra pars compacta connections (Marani et al., 2008) and thereby changes local dopamine concentrations. Indeed, in studies in rodents and pigs it has been shown that STN DBS increases striatal dopamine (Meissner et al., 2003; Shon et al., 2010). And although results of studies on STN DBS on striatal dopamine remain controversial, Yamamoto et al showed a significant correlation between STN activity and striatal monoamine concentrations in the normal and the PD rat (Yamamoto et al., 2013). We therefore hypothesized that STN DBS in humans would alter striatal dopamine and could influence glucose metabolism via dopaminergic pathways. However, whether striatal dopamine increases upon STN DBS in humans is unclear. Hilker et al could not demonstrate an increase in striatal dopamine release in Parkinson patients with STN DBS using positron emission tomography with the dopamine D2/3-receptor ligand [^11^C]raclopride (Hilker et al., 2008). Although, it is known that with this technique, small changes in dopamine cannot be detected. Another explanation for the lack of a metabolic effect could be that the changes in striatal dopamine observed in animals cannot be directly translated to the human situation.

Finally, it could be that in our study, dopamine changes within the striatum were not extensive enough to alter glucose metabolism, and that there is a certain threshold needed to exert an effect. Indeed, pig studies showed a dose effect curve on dopamine release, which was intensity- and frequency- dependent (Shon et al., 2010). Our patients remained on their levodopa medication during both study days because they had clinical benefit from this treatment and the medication could not be discontinued on ethical grounds. This might have disguised any additional effects of the STN DBS and we cannot fully exclude possible influences of their medication, however, to minimize possible interference with our study results, the patients took their medication at the same time points on both study days, and the patients served as their own controls. It is not known whether the earlier described beneficial effects of dopamine agonists on glucose metabolism in humans (Pijl et al., 2000) occur via central or peripheral mechanisms.

It has been proposed that gamma-aminobutyric acid (GABA) could also mediate the beneficial effects on locomotor symptoms of STN DBS in Parkinson patients, because in rats stimulated with DBS in the STN, extracellular GABA is increased besides dopamine (Windels et al., 2000, 2003) and thus a combination of an increase in dopamine and GABA release might be responsible for the observed effects. It has been argued that this change in GABA is related to reducing of pathological hyperactivity in output structures of the STN. There is however no evidence that this would also result in changes in activity in hypothalamic areas involved in the regulation of glucose metabolism. It is clear from our data though, that whether dopamine and/or GABA are altered in Parkinson patients treated with STN-DBS (or not), stimulation does not result in acute changes in glucose metabolism.

In our study, we measured acute effects of STN DBS on glucose metabolism and insulin sensitivity. Long-term effects of DBS STN on these parameters are unknown. It has been suggested that PD in itself is associated with the occurrence of diabetes (Skeie et al., 2013). Studies show that patients with PD display a dopaminergic dysfunction in the hypothalamus (Politis et al., 2008), which might contribute to the development of endocrine disorders associated with this disease. The occurrence of type II diabetes in patients with DBS of the STN however, is difficult to interpret, because most of these patients gain weight/fat mass after placement of the DBS (Barichella et al., 2003; Montaurier et al., 2007; Bannier et al., 2009), which might disguise any favorable effect of the DBS itself. Furthermore, most patients also decrease their levodopa medication after DBS placement.

## Conclusion

DBS of the STN in patients with PD does not have an acute effect on basal glucose metabolism nor on insulin sensitivity. These results suggest that modulation of the basal ganglia circuitry does not affect glucose metabolism in humans. Part of the lack of the effect might be explained by the concurrent use of levodopa medication.

## Author contributions

Nicolette M. Lammers, Brigitte M. Sondermeijer, Th. B. (Marcel) Twickler, Rob M. de Bie, Susanne E. La Fleur, and Mireille J. Serlie contributed to the conception and design of the study. Nicolette M. Lammers and Brigitte M. Sondermeijer were responsible for data acquisition, Nicolette M. Lammers, Susanne E. La Fleur, and Mireille J. Serlie contributed to the data analysis. All authors contributed to the interpretation of the data and preparation of the manuscript. All authors approve the version that is currently under consideration and acknowledge that they are accountable for all aspects of the work.

### Conflict of interest statement

P. Richard Schuurman is an independent consultant for Medtronic Inc. on educational matters and received travel grants from the company. Rob M. de Bie received a fellowship grant from Medtronic. All other authors report no conflicts of interest.
